# URA3 as a Selectable Marker for Disruption and Functional Assessment of *PacC* Gene in the Entomopathogenic Fungus *Isaria javanica*

**DOI:** 10.3390/jof9010092

**Published:** 2023-01-08

**Authors:** Manling Zou, Bei Xin, Xin Sun, Runmao Lin, Junru Lu, Jing Qi, Bingyan Xie, Xinyue Cheng

**Affiliations:** 1 College of Life Sciences, Beijing Normal University, Beijing 100875, China; 2 Institute of Vegetables and Flowers, Chinese Academy of Agricultural Sciences, Beijing 100081, China; 3 Ministry of Education Key Laboratory for Biodiversity Science and Ecological Engineering, Beijing 100080, China

**Keywords:** entomopathogenic fungus, *Isaria javanica*, *ura3* gene, uridine auxotrophy, markerless transformation, *PacC* gene, the pH response transcription factor

## Abstract

An effective selection marker is necessary for genetic engineering and functional genomics research in the post-genomic era. *Isaria javanica* is an important entomopathogenic fungus with a broad host range and prospective biocontrol potentials. Given that no antibiotic marker is available currently in this fungus, developing an effective selection marker is necessary. In this study, by applying overlap PCR and split-marker deletion strategy, combining PEG-mediated protoplasm transformation method, the uridine auxotrophy gene (*ura3*) in the *I. javanica* genome was knocked out. Then, using this transformation system, the pH response transcription factor gene (*IjpacC*) was disrupted successfully. Loss of *IjpacC* gene results in an obvious decrease in conidial production, but little impact on mycelial growth. The virulence of the Δ*IjpacC* mutant on caterpillars is similar to that of the wild-type strain. RT-qPCR detection shows that expression level of an acidic-expressed S53 gene (IF1G_06234) in Δ*IjpacC* mutant is more significantly upregulated than in the wild-type strain during the fungal infection on caterpillars. Our results indicate that a markerless transformation system based upon complementation of uridine auxotrophy is successfully developed in *I. javanica*, which is useful for exploring gene function and for genetic engineering to enhance biological control potential of the fungus.

## 1. Introduction

*Isaria javanica* is an insect pathogenic fungus belonging to the family Cordycipitaceae (Ascomycota: Sordariomycetes: Hypocreales) [1], which is widely distributed in the world. Previous studies have proved that this fungus has high virulence to insects in Lepidoptera, such as caterpillars of *Phthorimaea operculella* [2], *Lymantria dispar* [3], *Duponchelia fovealis* [4], etc.; in Hemiptera, such as psyllid *Diaphorina citri* [5,6], whitefly *Bemisia tabaci* [7,8,9], aphids *Hyalopterus pruni*, *Aphis pomi* and *Myzus persicae* [10,11], leafhopper *Empoasca vitis* [12]; in Thysanoptera, such as thrip *Thrips palmi* [13]; in Hymenoptera, such as ant *Solenopsis invicta* [14]; in Isoptera, such as termite *Coptotermes gestroi* [15], and so on. Because of its wide host range and strong virulence, *I. javanica* is considered to have great biocontrol potentials. So far, the genome sequence of *I. javanica* has been published [16]. Functional genomics research and genetic engineering will be hot topics in the post-genomic era.

In the process of genetic modification of an organism, selection markers are essential to effectively detect and screen transformants [17]. Antibiotic selection markers are commonly used in fungal gene knockout. However, through an antibiotic sensitivity test, we found that the fungus *I. javanica* is not sensitive to both hygromycin and geneticin (Supplemental Figure S1), which are two commonly used antibiotic-selective markers in fungal transformant screening. This indicates that the fungus *I. javanica* has intrinsic resistance to antibiotic-selective markers, rendering these agents unsuitable for use in transformation systems for this fungus. Auxotrophy is another type of selection marker, which does not require the use of antibiotics and is commonly used in filamentous fungi transformation. Uridine auxotrophy gene (*ura3*) is one of the widely used auxotrophy selection markers. It encodes an orotidine 5′-phosphate decarboxylase (OMP decarboxylase) and plays an important role in the uracil synthesis pathway. If 5′-fluoroorotic acid (5-FOA) is added to the culture medium, the fungus (wild-type) fails to grow normally on the medium because 5-FOA can be transformed into toxic 5′-fluorouracil [18]. After *ura3* gene is knocked out, the mutant loss its sensitive to 5-FOA and can only grow on the medium containing exogenous uracil or exogenous uridine [19,20]. Thus, *ura3* gene can be used as a marker for genetic manipulation. So far, auxotrophic strains have been acquired in a variety of fungi, such as *Aspergillus*, *Trichoderma*, *Mortierella,* and *Beauveria*. The use of uridine auxotrophy has been widely exploited for the development of genetic transformation system in many filamentous fungi [21,22,23,24]. These studies provide references for establishing a *ura3*-based marker recycling system in *I. javanica*, which is necessary for genetic operation in the fungus.

In this study, by using homologous recombination methods, we first knocked out the *Ijura3* gene and established a uridine auxotrophic genetic operation system in *I. javanica*. Then, we selected the pH response transcription factor PacC/Rim101 gene (*IjpacC*) as a target, which can activate alkaline-expressed genes and repress acid-expressed genes under neutral to alkaline conditions, and plays important roles in fungal development, reproduction, and pathogenesis [25,26]. We tested the usability of the established markerless transformation system and explored the function of *PacC* in *I. javanica*.

## 2. Materials and Methods

### 2.1. Fungal Strains and Growth Conditions

Two *I. javainica* strains were used to construct Δ*Ijura3* mutants in parallel. One is the strain Pj01, which was isolated from the oriental leafworm *Spodoptera litura* and deposited into the China Center for Type Culture Collection (No. CCTCCM208107). Another is the strain IJB01, which was isolated from whitefly *Bemisia tabaci* and deposited into the China General Microbiological Culture Collection Center (CGMCC) with the number CGMCC3.18094 [16]. Both strains were cultured on potato dextrose agar (PDA) at 25 °C in an incubator.

### 2.2. Extraction of Genomic DNA, Total RNA, and cDNA Synthesis

Conidial spores of the fungus were cultured in potato dextrose broth (PDB) at 25 °C with shaking at 150 rpm for 5 days. Then, fresh mycelium tissue was harvested by filtration and used for DNA and RNA extraction. Genomic DNA was isolated from freeze-dried mycelium using a regular phenol chloroform method. The total RNA was isolated from freeze-dried mycelium using TRIzol reagent (Invitrogen, Carlsbad, CA, USA). The concentration and purity were measured with an ultraviolet spectrophotometer, and the RNA integrity was checked by agarose gel electrophoresis. Then, the first-strand cDNA was synthesized using a FastQuant RT Kit (With gDNase) (Tiangen Biotech, Beijing, China), according to the manufacturer’s instruction.

### 2.3. PCR and RT-PCR

Using genomic DNA or cDNA as template, PCR or RT-PCR reaction was performed in 25 or 50 µL volume using Phata Taq Polymerase (Vazyme Biotech, Nanjing, China), with an initial denaturation at 95 °C for 3 min, 35 cycles of 95 °C for 15 s, Tm °C for 15 s, and 72 °C for X s (set up as 30 s/kb), and finally, elongation at 72 °C for 5 min. PCR product was purified with the Easy Pure Quick Gel Extraction Kit (Vazyme Biotech, Nanjing, China), according to manufacturer’s protocol.

### 2.4. Genetic Manipulations for Ijura3 Gene Knockout and Complementation

We applied overlap PCR and the split-marker deletion strategy [27,28,29] to knock out *Ijura3* gene (Supplemental Figure S2a). The coding gene of hygromycin B phosphotransferase (*hph*) was used to substitute the *Ijura3* gene. Experiment details are as follows: first, using *I. javanica* genomic DNA of both strain Pj01 and strain IJB01 as templates, fragments of ~1.43 kb of the upstream and ~1.25 kb of the downstream nucleotide sequences of *Ijura3* were amplified by PCR amplification with the primer pairs of UF/UR and DF/DR, respectively. Using the plasmid pKOV21 that contains the *hph* gene as template, the fore-2/3 and the post-2/3 fragments of *hph* gene were amplified with the primer pairs of HUF/H1R and H2F/HDR, respectively. Then, taking the amplified fragments of the upstream sequence of *Ijura3* and the fore-2/3 *hph* gene as a template, by overlap PCR amplification with the primer pair of UF/H1R, a fragment containing the upstream sequence of *Ijura3* and the fore-2/3 of *hph* was produced. Taking the fragments of the downstream sequence of *Ijura3* and the post-2/3 *hph* gene as templates, PCR amplification with the primer pair of H2F/DR, a fragment containing the post-2/3 *hph* and the downstream sequence of *Ijura3* was produced. The PCR products were purified with an EasyPure^®^ Quick Gel Extraction Kit (Transgen, Beijing, China), according to manufacturer’s protocol. The purified DNA was used for transformation. All primers used in this study are listed in supporting Table S1. Moreover, with the primer pair of UF/DR, a length of about 4 kb DNA fragment containing the *Ijura3* gene along with its upstream and downstream sequences was also amplified from genomic DNA of *I. javanica* strain Pj01. Then, it was purified and used for transformation and *ura3* complementation in the mutants of strain Pj01 (Supplemental Figure S2b).

### 2.5. Preparation of Protoplasts

Conidia (~5 × 10^8^ spores) was cultured in 200 mL PDB at 25 °C for 12–14 h, with shaking at 150 rpm. Freshly germinated conidial pellets were collected after filtered through four layers of sterile filter paper and washed thoroughly with 0.7 M NaCl. Then, 1 g mycelium was digested with 2 mL of Yatalase enzyme (20 mg/mL) (Takara, Dalian, China) at 28 °C for 3–4 h, with shaking at 120 rpm. Then, STC buffer (0.7 M sorbitol, 50 mM CaCl_2_•2H_2_O, 10 mM Tris/HCl, pH 7.5) was added to stop reaction (at 0 °C). The enzymatic hydrolysate was filtered through four layers of sterile filter paper, rinsed repeatedly with STC buffer, and centrifuged at 3000 rpm for 15 min to collect protoplasts. Then, the protoplast deposit was resuspended with STC buffer, and the concentration was adjusted to 2 × 10^8^/mL. The prepared protoplasts were directly used for subsequent transformation.

### 2.6. PEG-Mediated Protoplast Transformation

About 5 µg DNA (the purified PCR products) was added to 10 µL aurintricarboxylic acid (100 mM), quantified to 60 µL with TEC buffer (10 mM Tris-Cl, pH 7.5, 1 mM EDTA, 40 mM CaCl_2_•H_2_O), and incubated on ice for 20 min. After centrifugation with 12,000 rpm for 2 min, the supernatant was added into 100 µL protoplasts and incubated on ice for 20 min. Then, 160 µL PEG was added to the protoplast mixture and incubated at room temperature for 15 min. A total of 1 mL STC (40 mM CaCl_2_•2H_2_O, 1 mM EDTA, 10 mM Tris-Cl, pH 7.5) was added to stop the incubation, and then the mixture was centrifuged with 3000 rpm for 5 min. The protoplasts were isolated and resuspended with 200 µL STC. Then, 50 µL of transformed protoplasts was added to 10 mL MM medium containing 0.5 mg/mL uridine, and the mixture was coated on MM agar plates (preheated at 37 °C) and cultured at 25 °C after solidification. After 12–20 h, 5 mL T-top containing 1 mg/mL 5- FOA was laid on the transformation plate and cultured upside down at 25 °C, until hyphae grew on the surface of T-top. Then, they were picked up on a new PDA plate containing 0.5 mg/mL uridine and 1 mg/mL 5-FOA to obtain single colonies. At the same, *ura3* complements were screened out, except for the absence of the uridine and 5-FOA in MM and T-Top media. Further verification of positive transformants were performed.

### 2.7. Verification of Transformants

Positive transformants were verified by PCR, RT-PCR, and Southern blot methods. Selected transformants were grown in liquid PDB medium containing 0.5 mg/mL uridine and 1 mg/mL 5-FOA at 25 °C for 3 days with 150 rpm sharking. For screening *ura3* complementary transformants, no uridine and 5-FOA were contained in all media. Mycelium was collected and used for genomic DNA and total RNA extraction. Then, using genomic DNA as templates, PCR amplification was performed with primer pairs of UF/UR, DF/DR, H1F/H2R, and ura3F/ura3R to detect the upstream and downstream of *ura3*, *hph,* and *ura3* gene. Using cDNA as templates, RT-PCR amplification was performed with primer pair of RTU_F/ RTU_ R to detect expression of *ura3* gene. Expression of β-tubulin gene was taken as control, with primer pair of tubulin_F/tubulin_R. PCR products were detected by agarose gel electrophoresis.

Southern blot was performed to detect *Ijura3* gene deletion. About 40–60 µg of genomic DNA from each transformant was digested with the restriction endonuclease *Nae* I at 37 °C overnight. Then, DNA fragments were separated on 0.8% agarose gel by electrophoresis, and subsequently transferred to nitrocellulose hybrid membrane using standard protocols. Blots were probed using a 547 bp PCR product that was amplified from the fungal genome with primer pair TAN2_F/R, which was within the upstream flanking sequence of the *ura3* gene. Probe preparation, membrane hybridization, and visualization were performed using a DIG-High Prime DNA Labeling and Detection Starter Kit (Roche, Penzberg, Geermany), according to manufacturer’s protocol.

### 2.8. IjpacC Gene Disruption by Using Uracil Anxotrophy Transformation System

The pUC19 vector was used to construct *IjpacC* knockout vector (Supplemental Figure S2c). Using the wild-type genomic DNA of *I. javanica* strain Pj01 as template, PCR amplification was performed with primer pair PUF/PUR to amplify the upstream sequence of *IjpacC* gene (~1.5 kb), with PDF/PDR to amplify the downstream sequence of *IjpacC* gene (~1.5 kb), and with Pura3F/Pura3R to amplify the open reading frame (ORF) of *ura3* gene (~1.1 kb). Then, the three fragments (purified PCR products) were connected to the pUC19 vector with the ClonExpress MultiS One Step Cloning Kit (Vazyme, Nanjing, China), according to manufacturer’s protocol. The constructed vector was then transferred into protoplasts of **Δ***Ijura3* mutants of the strain Pj01 by PEG-mediated protoplast transformation, as above described. The screening of **Δ***IjpacC* mutants was performed in the same way as screening of the *ura3* complementary transformants, with no uridine and 5-FOA contained in all media. Positive transformants were further determined by PCR with primer pairs of PacCF/PacCR, Pura3F/Pura3R, PUF/PUR, and PDF/PDR to amplify *IjpacC* and *Ijura3* upstream and downstream of *PacC* gene, respectively. RT-PCR was performed with primers RTU_F/R and RTP_F/R to detect gene expression of *ura3* and *PacC*, respectively. Expression of *β-tubulin* gene was taken as control for RT-PCR.

### 2.9. Growth and Conidiation Assays

Vegetative growth: The wild-type and the *ura3* complementation (**Δ***Ijura3::ura3*) strains were inoculated in PDB medium, and the **Δ***Ijura3* mutant strains were inoculated in PDB medium containing 0.5 mg/mL uridine and 1 mg/mL 5-FOA, and then cultured at 25 °C for 5 days with shacking at 150 rpm. Then, the conidial spores were filtered and adjusted to a concentration of 1 × 10^6^ conidia/mL. Then, 1 µL of each conidial suspension was inoculated on a plate of PDA, PDA containing 0.5 mg/mL uridine (PDAU), or PDAU plus 1 mg/mL 5-FOA. After growth at 25 °C for 7 days, the colony diameter of each plate was measured through the crossing method. For assay, the growth rate of Δ*IjpacC* mutant, the wild-type, and the Δ*IjpacC* mutant strains were cultured on PDA at neutral condition (pH = 7), and the diameter of each colony was measured by crossing the method every 2 days. Each strain had three repetitions, and all experiments were repeated three times.

Conidial production: To test conidial production of each strain, a 100 µL of conidial suspension (~10^5^ spores) was coated on a 90 mm PDAU plate and cultured at 25 °C for 7 days. Then, two pieces of mycelium with 10 mm diameter were taken from each plate, and then homogenized in 3 mL water containing 0.01% Tween 20. Spore numbers were counted with a hemocytometer and results presented as spores per square centimeter of agar surface [30]. Each strain had three repetitions, and experiments were repeated three times. To test the influence of *IjpacC* deletion on conidial production, conidial suspensions of the wild-type and Δ*IjpacC* mutant strains were coated on PDA plates with three different pH values (pH 5.0, 7.0 and 11.0) and cultured at 25 °C for 7 days. The number of the spores was counted as above. Each strain had three repetitions, and all treatments were repeated three times.

### 2.10. Insect Bioassay

We used the potato tuberworm *Phthorimaea operculella* (Lepidoptera) to assay fungal virulence change after deletion of the *IjpacC* gene of the fungus *I. javanica*. A stable population of *P. operculella* was maintained in the laboratory and reared on potato tubers in an insect-rearing chamber at 27 ± 0.5 °C with a photoperiod of 12L:12D, according to the method described in a previous report [2]. A 10 μL of conidial suspension (1 × 10^8^ conidia/mL) of Δ*IjpacC* mutant or WT strain was dripped on each 3rd or 4th instar larva of the potato tuberworm, and 30 larvae were treated for each strain. These larvae were placed in a 90 mm dish and reared on potato chips at 27 °C. Treatment with sterile water was taken as control. The number of dead larvae was counted daily and the mortality was calculated. Each treatment had three repetitions, and experiment was performed with three biological repeats.

### 2.11. Quantitative RT-PCR

To explore the impact of *IjpacC* deletion on the expression of acidic-expressed S53 genes, a S53 gene (IF1G_06234) was taken as a target, which was reported to upregulate significantly during the fungal infection in insects [16]. The wild-type and Δ*IjpacC* mutant strains were inoculated on the 3rd or 4th instar larvae of the potato tuberworm, respectively. After infection for 24, 48, and 72 h, larvae were collected. Each treatment had three repetitions. Fungal strains without infection were taken as controls (0 h). Total RNA was extracted from samples of each treatment larvae and cDNA was synthesized. Using cDNA as template, qRT-PCR was performed to detect expression levels of the target fragment of the fungal S53 gene with the primer pair IF1G_06234F/R, by using a SYBR Premix ExTaq^TM^ II kit (Vazyme, Nanjing, China) and a BIO-RAD CFX96 PCR system (BIO-RAD, Hercules, CA, USA). All reactions were run in triplicate. Expression of actin gene was taken as the internal control. The changes in relative gene expression in different time periods were calculated by the 2^−ΔΔCT^ method [31]. By comparing the relative expressions of the target gene (IF1G_06234F/R) in Δ*IjpacC* mutant and the wild-type strains, the impact of *IjpacC* deletion on S53 gene expression was observed.

### 2.12. Data Analysis

For data statistical analysis, we first tested the data with leveneTest for homogeneity of variance and Shapiro–Wilk normality test for Gaussian distribution. Then, *t*-test and analysis of varience (ANOVA) were carried out. ANOVA was conducted via the aov () function. A non-parametric test was carried out via the Kruskal–Wallis method and multiple comparisons by using the ‘wmc’ function (https://www.statmethods.net/RiA/wmc.txt, accessed on 22 June 2022). All data analyses were performed in R 4.2.1 [32] and data visualization was conducted in Origin software (version 2022b, https://www.originsoftware.net/, accessed on 20 August 2022).

## 3. Results

### 3.1. Generation of Ijura3 Mutant and Complementation Strains

**Figure 1:**
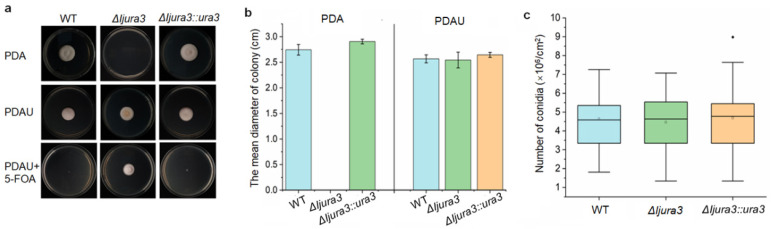
Comparison of cultural traits among the wild-type, *Ijura3* mutant, and complementation (Δ*Ijura3::ura3*) strains of *I. javanica*. (**a**) Colony grown on PDA, PDA plus 0.5 mg/mL uridine (PDAU), and PDAU plus 1 mg/mL 5-FOA. (**b**) Colony sizes of different strains on PDA and PDAU cultured at 25 °C for 7 days. Error bar represents the standard error of the mean. (**c**) Conidial production on PDAU after culture at 25 °C for 7 days. The marker (*) in Box-plot represents outlier.

Through two round PCR amplification, we acquired two fusion fragments with the size 2.5 kb and 2 kb, respectively (Supplemental Figure S3a). Then, by PEG-mediated protoplast transformation and homologous recombination, the *ura3* gene was successfully replaced by *hph* gene. The auxotrophic mutant (Δ*Ijura3*) strains of *I. javanica* can grow on PDA containing 0.5 mg/mL uridine and 1 mg/mL 5- FOA, but not on a normal PDA (Figure 1a). The mutant strains were further confirmed by PCR amplification of fragments of *hph* and *ura3*, upstream and downstream of *ura3* genes (Supplemental Figure S3b). Total RNA was extracted (Supplemental Figure S3c) and RT-PCR was performed to detect expression of *ura3* gene (Supplemental Figure S3d). Southern blot analysis shows that, in the *Ijura3* mutant sample, a fragment with ~4000 bp in size is displayed, which includes the upstream and downstream sequences of the *ura3* gene and the *hph* gene. While in the wild-type sample, a fragment with ~2660 bp in size is displayed, which includes the *ura3* gene and its flanking sequences (Supplemental Figure S3e).

For *ura3* gene complementation, the full length of *Ijura3* gene with upstream and downstream flanking sequences (~4.1 kb) was amplified from the WT strain of *I. javanica*. Then, by homologous recombination, the *ura3* gene was complemented into the Δ*Ijura3* mutant genome, and complementation strains (Δ*Ijura3::ura3*) were obtained. As the wild-type strain, the complementation strains can grow on normal PDA, but not on PDA with 0.5 mg/mL uridine and 1 mg/mL 5-FOA (Figure 1a). Transformants were further confirmed by PCR (Supplemental Figure S3f) and RT-PCR (Supplemental Figure S3g).

### 3.2. No Influence on Growth Rate and Conidial Production after Deletion of Ijura3 in I. javanica

Vegetative growth and conidial production were examined and compared among the wild-type, Δ*Ijura3,* and Δ*Ijura3*::*ura3* strains. As the Δ*Ijura3* strain cannot grow on PDA medium, we compared their growth rates on PDA medium supplemented with 0.5 mg/mL uridine (PDAU), which all strains can grow on (Figure 1a). After being cultured at 25 °C for 7 days, the mean colony diameter of the wild-type strain is 2.57 cm, that of the Δ*Ijura3* strain is 2.54 cm, and of the *ura3* complementation strain is 2.64 cm. Statistical analysis shows that the differences among them are not significant (*df* = 2, *F* = 1.648, *p* = 0.439) (Figure 1b). Their colony morphology is also similar, with no obvious difference among these strains (Figure 1a). We also measured the colony sizes of the wild-type and the complementation strains grown on PDA, and the mean diameters are 2.74 cm and 2.91 cm, respectively. The difference between the two strains is also not significant at a statistical level by *t*-test (*df* = 16, *t* = −1.422, *p* = 0.174) (Figure 1b).

We also compared the conidiation of different strains on PDAU. After growth for 7 days, the mean spore yield of the wild-type strain is 4.63 × 10^6^ conidia/cm^2^, while that of the Δ*Ijura3* strain is 4.46 × 10^6^ conidia/cm^2^, and of the Δ*Ijura3::ura3* strain is 4.69 × 10^6^ conidia/cm^2^. ANOVA analysis shows no obvious difference among them (*df* = 2, 51; *F* = 0.088, *p* = 0.916) (Figure 1c). The results indicate that deletion of *Ijura3* gene has no impacts on mycelial growth and conidial production in *I. javanica*, suggesting that *ura3* gene is available as a selective marker for genetic operation in the fungus.

### 3.3. Deletion of IjpacC Gene Using URA3 as a Selection Marker

**Figure 2:**
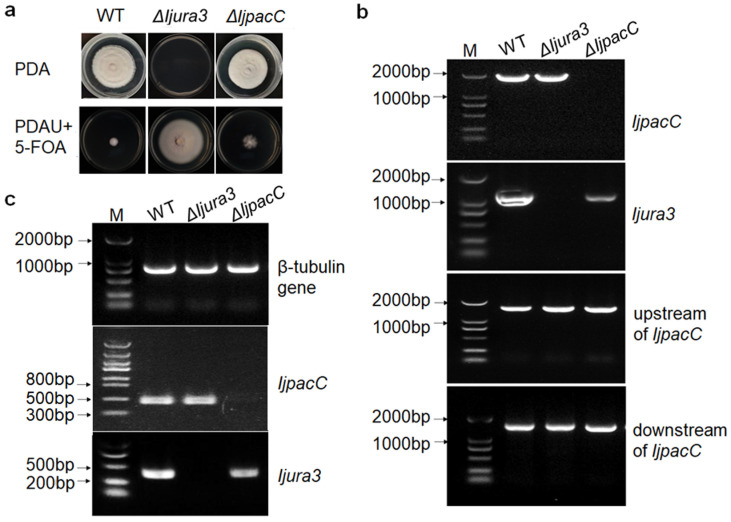
Verification of *PacC* gene deletion in *I. javanica.* (**a**) Colony of the wild-type, *Ijura3* mutant, and *IjpacC* mutant strains grown on PDA and PDA with 0.5 mg/mL uridine and 1 mg/mL 5-FOA at 25 °C for 20 days. (**b**) PCR detection of different fragments. (**c**) RT-PCR detection of gene expressions of *IjpacC* and *Ijura3* in different strains, with expression of *β-tubulin* gene as control.

For deletion of *IjpacC* gene, three fragments (upstream and downstream sequences of *PacC* gene, and the ORF of *ura3* gene) were inserted into the pUC19 vector to generate the gene replacement plasmid pUC19-*IjpacC*. Then, it was transferred into the protoplast of **Δ***Ijura3* mutant strain by PEG-mediated protoplast transformation. Gene disruption was achieved via homologous recombination and *IjpacC* gene was replaced by the *ura3* gene (Supplemental Figure S2c). The correct recombinant can grow on PDA medium, but not on PDA with 0.5 mg/mL uridine and 1 mg/mL 5-FOA (Figure 2a). PCR and RT-PCR detection also shows that the *PacC* gene is deleted (Figure 2b) and cannot be expressed (Figure 2c) in the **Δ***IjpacC* mutant strain, while the *ura3* gene is complemented and expressed (Figure 2b,c). The result indicates that using the URA3 auxotrophic knockout system target gene can be successfully knocked out in the fungus *I. javanica*.

### 3.4. Disruption of IjpacC Gene May Affects Conidial Yield in I. javanica

**Figure 3:**
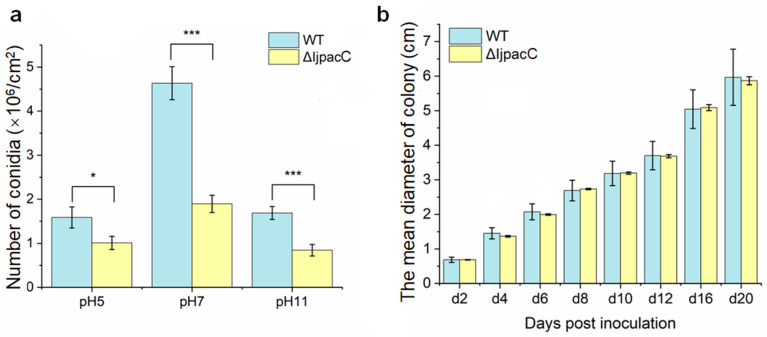
Comparison of conidial production and hyphal growth between the Δ*IjpacC* mutant and the wild-type strains. (**a**) Conidiation under different pH conditions. (**b**) Vegetative growth under neutral condition (pH7). Fungal strains were cultured on PDA at 25 °C for 7 days. Error bar represents the standard error of the mean. Asterisk marker represents significant difference at a statistical level, (*) *p* < 0.05, (***) *p* < 0.001.

We examined conidial spore yields of Δ*IjpacC* mutant strain under different pH conditions (pH = 5, 7, 11), and compared them with those of the wild-type strain. The result shows that, after growth on PDA at 25 °C for 7 days, the conidial yields of the two strains are different. Under acidic condition (pH5), the mean spore yield of the **Δ***IjpacC* strain is 1.01 × 10^6^ conidia/cm^2^, while that of the wild-type strain is 1.59 × 10^6^ conidia/cm^2^. The difference between them is significant at a statistical level (*df* = 34, *t* = −2.039, *p* = 0.049). Under neutral condition (pH7), the mean spore yield of the **Δ***IjpacC* strain is 1.89 × 10^6^ conidia/cm^2^, which is significantly less than that of the wild-type strain (4.63 × 10^6^ conidia/cm^2^) (*df* = 25.53, *t* = −6.471, *p* < 0.001). Under alkaline condition (pH11), the spore yield of the **Δ***IjpacC* strain is 0.84 × 10^6^ conidia/cm^2^, but the wild-type strain is 1.69 × 10^6^ conidia/cm^2^. The difference is also significant statistically (*df* = 34, *t* = −4.275, *p* < 0.001). Obviously, by disruption of *IjpacC* gene the conidial spore production is reduced in *I. javanica*, whether under acidic, neutral, or alkaline conditions. Moreover, under neutral condition (pH = 7), both the Δ*IjpacC* and wild-type strains can produce more conidial spores than those under acidic (pH = 5) and alkaline (pH = 11) conditions (Figure 3a).

We further compared the hyphal growth rates of the Δ*IjpacC* mutant and the wide-type strains on PDA under neutral condition (pH = 7). The result shows that the mean colony diameter of the Δ*IjpacC* mutant is similar to or slightly less than that of the wild-type strain. There is no significant difference between the two strains (Figure 3b). Our results indicate that deletion of *IjpacC* gene can impact on conidial production, but with minimal impact on hyphal growth in the fungus *I. javanica*.

### 3.5. Effect of IjpacC Deletion on Virulence of the Fungus to Caterpillars

**Figure 4:**
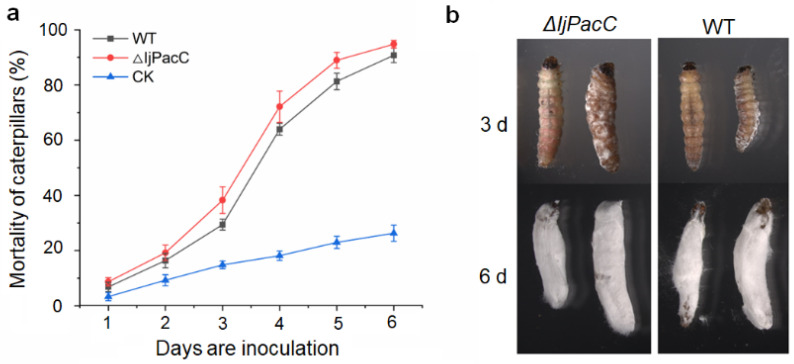
Virulence of the Δ*IjpacC* mutant and wild-type strains of *I. javanica* on the potato tuberworm. (**a**) Mortality curve of caterpillars infected with different *I. javanica* strains. CK: control, caterpillars were treated with sterile water. (**b**) Fungus parasites on caterpillars after inoculation for 3 and 6 days. Error bar represents the standard error of the mean.

We examined the effects of *IjpacC* deletion on virulence of the fungus to the 3rd or 4th instar larvae of the potato tuberworm. By observation of the mortality of larvae daily after inoculation of a 10 μL of spore suspension (1 × 10^8^ conidia/mL) on each individual, the result shows that the mean mortality of caterpillars infecting with **Δ***IjpacC* strain is slightly larger than that of those infecting with the wild-type strain (Figure 4a), but the difference between them is not significant (*p* > 0.05). After inoculation for four days, the correct mortalities of both strains are larger than 50%. After inoculation for six days, the correct mortalities of both are nearly 90% (Figure 4b). The result indicates that deletion of *IjpacC* gene has no obvious influence on the fungal virulence to caterpillars.

### 3.6. Influence of IjpacC Deletion on Expression of Acidic-Expressed Gene

**Figure 5:**
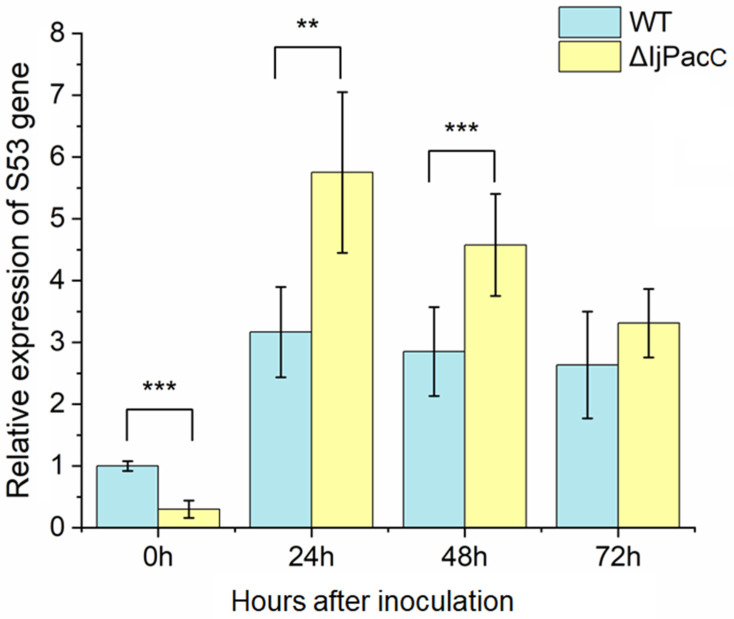
Expression dynamics of a S53 gene (IF1G_06234) in the Δ*IjpacC* mutant and WT strains of *I. javanica* during the fungal infection on caterpillars. Error bar represents the standard error of the mean. Asterisk marker represents significant difference at a statistical level, (**) *p* < 0.01, (***) *p* < 0.001.

To determine impacts of *IjpacC* deletion on acidic-expressed gene, we detected the expression dynamics of a S53 gene (IF1G_06234), which was reported to be obviously upregulated during the infection process of *I. javanica* parasitizing caterpillars of the potato tuberworm [16]. By RT-qPCR detection, it is shown that the expression levels of the S53 gene in both strains (WT and **Δ***IjpacC*) are obviously upregulated during the fungal infection process compared with those before inoculation (0 h) (Figure 5). Notably, the upregulation is more significant in the **Δ***IjpacC* mutant than in the wild-type strain, especially at the early infection stage. At 24 h after inoculation, the expression level in **Δ***IjpacC* mutant is significantly higher than that in the WT strain (*df* = 16, *t* = 2.96, *p* = 0.001). Moreover, at 48 h and 72 h after inoculation, the expression levels in the mutant strain are higher than those in the WT strain, and their differences are significant at statistic levels (for 48 h, *df* = 16, *t* = 5.175, *p* < 0.001; for 72 h, *df* = 16, *t* = 2.054, *p* = 0.057). However, before infection (0 h), the expression level of the S53 gene in the **Δ***IjpacC* mutant is obviously less than that in the WT strain (*t* = −4.22, *df* = 15, *p* < 0.001). The result indicates that deletion of *IjpacC* gene can increase expression of the S53 gene during the fungus infection on caterpillars.

## 4. Discussion

Entomopathogenic fungi play an important role in the biological control of insect pests. Genetic manipulation can improve pathogenic fungi and make their characters more conducive to biological control in the field, such as improving the fungal virulence. Most genetic modification depends on antibiotic markers. However, no antibiotic marker is available in the fungal *I. javanica* currently. In this study, by overlapping PCR and the split-marker deletion strategy, and combining PEG-mediated protoplasm transformation method, we successfully knocked out the *Ijura3* gene in the *I. javanica* genome, which can be screened by 5-FOA (Figure 1a). The knockout of *Ijura3* gene is independently performed in the two *I. javanica* strains, indicating that the transformation is rather robust and not strain-dependent. By comparison, both the hyphal growth and conidial production were detected significant difference among the wild-type, Δ*Ijura3* mutant, and complementation strains (Figure 1b,c), indicating *ura3* gene as a selection marker is suitable for transformant screen in the fungus *I. javanica*. Utilizing the Δ*Ijura3* mutant strain, by homologous recombination, the *IjpacC* gene was successfully knocked out and **Δ***IjpacC* mutant strain was obtained (Figure 2). Our results suggest that a markerless knockout system is successfully established in the fungus *I. javanica*, which provides a useful approach for exploring gene function and for genetic manipulation. Of course, further improvement and optimization in approach are needed for increasing transformation frequency in future research.

Although the pH responsive transcription factor PacC/Rim101 is documented to play important roles in fungal development, reproduction, pathogenesis, and so on [25,26], the detail effects are species-specific. Loss of *PacC* gene often leads to damage of spore formation and spore germination, such as in *Aspergillus carbonarius* [30], *Magnaporthe oryzae* [25], etc. However, in *Aspergillus ochraceus*, loss of *PacC* gene can increase sporulation under both acidic and alkaline conditions [33]. *PacC* mutants show a reduced virulence in most phytopathogenic fungi, such as in *Botrytis cinerea* [34], *Clonostachys rosea* [35], etc. However, in the genus *Fusarium*, the *PacC* mutants show enhanced virulence [36]. While, in *Ustilago maydis*, *PacC* deletion shows no influence on the virulence [37]. In entomopathogenic fungi, such as in *Metarhizium acridum* and *M. robertsii*, *PacC* is vital for conidial yield, and conidiation is seriously impaired in *PacC* mutants. Deletion of *PacC* gene reduces the pathogenicity of *M. robertsii*, *M. acridum,* and *M. rileyi*, indicating *PacC* gene is necessary for the full virulence in these fungi [38,39,40]. However, in *Beauveria bassiana*, deletion of *PacC* gene only has minor impact on its virulence to larvae of *G. mellonella* (Lepidoptera) and *T. molitor* (Coleoptera) [41]. Nevertheless, in our study, deletion of *PacC* gene in *I. javanica* shows a reduced conidial yield, and little impact on hyphal growth rate (Figure 3), but a slightly increased virulence on caterpillars (Figure 4). Moreover, deletion of *IjpacC* gene can obviously increase expression level of an acidic-expressed gene during the fungal infection process (Figure 5). In the light of S53 serine peptidases particularly abundant in the *I. javanica* genome (with 19 members) [16], more work is necessary in future study for exploring *IjpacC* gene in regulation on expression of S53 genes. Furthermore, consideration of no information on potential ectopic plasmid integration events in this study, it is suggested to perform Southern blots using ura3 and vector backbone sequences as probes, and complementation of *pacC* gene to generate complementation strain (**Δ***IjpacC::pacC*). By which, phenotypic changes in **Δ***IjpacC* mutant caused by in combination with other recombination events may be excluded in future research.

## Data Availability

No new data were created.

## Supplementary Materials

The following supporting information can be downloaded at: https://www.mdpi.com/article/10.3390/jof9010092/s1, Supplemental Table S1. List of primers used in this study. Supplemental Figure S1. Sensitivity tests of *I. javanica* to hygromycin and geneticin (G418). Supplemental Figure S2. Flow chart of *ura3* and *PacC* gene knockout in *I. javanica*. a. *ura3* gene deletion. b. *ura3* complementation. c. *PacC* gene deletion. Supplemental Figure S3. Confirmation of constructs and transformants of Δ*Ijura3* mutant and Δ*Ijura3::ura3* complement. a. Electrophoresis detection of PCR products. b. PCR detection of positive transformants. c. Electrophoresis detection of RNA integrity. d. RT-PCR detection of positive transformants. e. Southern blot analysis. f. PCR detection of complements. g. RT-PCR detection of complements.
