# Supplementary material for: URA3 as a Selectable Marker for Disruption and Functional Assessment of PacC Gene in the Entomopathogenic Fungus Isaria javanica
Source: J Fungi (Basel). 2023 Jan 8;9(1):92. doi: 10.3390/jof9010092 (PMC9860623; doi:10.3390/jof9010092)
Supplement: Supplementary file 1 [file jof-09-00092-s001.zip › jof-2108620-supplementary.pdf]

Supplementary Table S1. List of primers used in this study

No.	Primer name	Sequence (5'-3')	Amplified Fragment	Tm (°C)	Size (bp)
1	UF	CTCGGTACGGTGAATAATGGC	The upstream sequence of *Ijura3* gene	57	1437
2	UR	acctccactagctccagccaaCCTCAGTCGCAAAGCACAGC		74	
3	DF	gaatagagtagatgccgaccggTCGTATGAGTGAGCTTGCCG	The downstream sequence of *Ijura3* gene	71	1254
4	DR	GTCGGATCGCTACTGCTACC		58	
5	HUF	gctgtgctttgcgactgaggcTTGGCTGGAGCTAGTGGAGGT	The upstream 2/3 fragment of *hph* gene	75	1114
6	H1R	GGATGCCTCCGCTCGAAGTA		60	
7	H2F	CGTTGCAAGACCTGCCTGAA	The downstream 2/3 fragment of *hph* gene	59	768
8	HDR	cggcaagctcactcatacgacCCGGTCGGCATCTACTCTATTC		71	
9	H1F	CTTGGCTGGAGCTAGTGGAGGT	Hygromycin resistance gene (*hph*)	61	1376
10	H2R	CCCGGTCGGCATCTACTCTATTC		60	
11	Ura3F	GTGGCTCACCCGACTCTCAAGGCG	*Ijura3* gene for verification	67	1034
12	Ura3R	GGATCATTTGCCTTGAGGATACCGCG		63	
13	PUF	aaacgacggccagtgaattcTTCATTTCTTGGTTGGTCTAGGC	The upstream sequence of *IjpacC* gene	69	1522
14	PUR	catGGCCGAAGTCGACGATAGG		61	
15	PDF	attgcgatgaGCATGGTACGCCGAGTGC	The downstream sequence of *IjpacC* gene	68	1530
16	PDR	accatgattacgccaagcttAGCTGCAGCTTTTGCCCC		71	
17	Pura3F	tatcgtcgacttcggccATGGTGGCTCACCCGACTC	*Ijura3* gene for substitution of *IjpacC* gene	72	1128
18	Pura3R	cgtaccatgcTCATCGCAATCTCTCCGTGTATG		66	
19	PacCF	GGTGAGCAGCAATAGCAGTGAC	*IjpacC* gene for verification	59	1848
20	PacCR	AAGAGCCAGGTACGGGAAGG		59	

21	tubulin_F	GGCGACGTATTCAATCGTTCCG	β-tubulin gene	60	792
22	tubulin_R	GCATCCTGATATTGCTGGTACTCGG		61	
23	RTU_F	CTCACCCGACTCTCAAGGCG	*Ijura3* gene for verification	61	368
24	RTU_R	GTGAGCCCAGTCAATGATGCG		60	
25	RTP_F	CGCAACTCCCCGACATCTCC	*IjpacC* gene for verification	61	501
26	RTP_R	GACCGGAACGCATCTGACCA		61	
27	IF1G_06947 F	GGACAACACGGCGTACCGGACC	Actin gene for qPCR	65	157
28	IF1G_06947 R	GCGCTCCTCGGCGTCGTCGG		69	
29	IF1G_02115F	CCAGCCGCCTCTGAACACCCC	PacC gene for qPCR	66	242
30	IF1G_02115R	GCGCCGGTTGAGGCATGTAGTG		64	
31	IF1G_06234F	GGCGAGAACAGGCAGGGCGAG	S53 gene for qPCR	66	274
32	IF1G_06234R	CGTATTCGCGCCGGAGAAGCTGC		66	
33	TAN2_F	GCCTGCAGTTGACCGATCCC	Probe for southern blot	62	547
34	TAN2_R	GTGAGGGTGTGACGAGTGTGCC		63	

Suppl. Fig. S1.

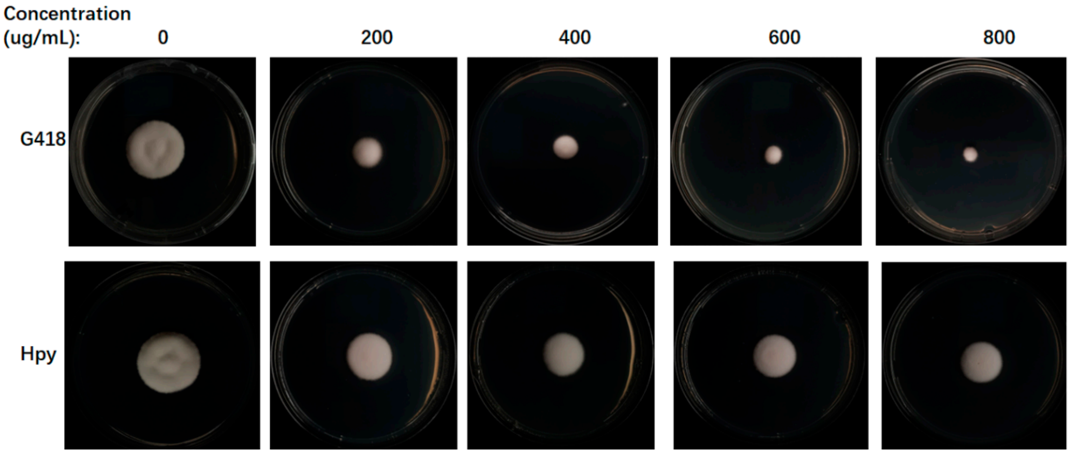

Suppl. Fig. S1. Sensitivity tests of *I. javanica* to hygromycin B (Hpy) and geneticin (G418)

Suppl. Fig. S2.

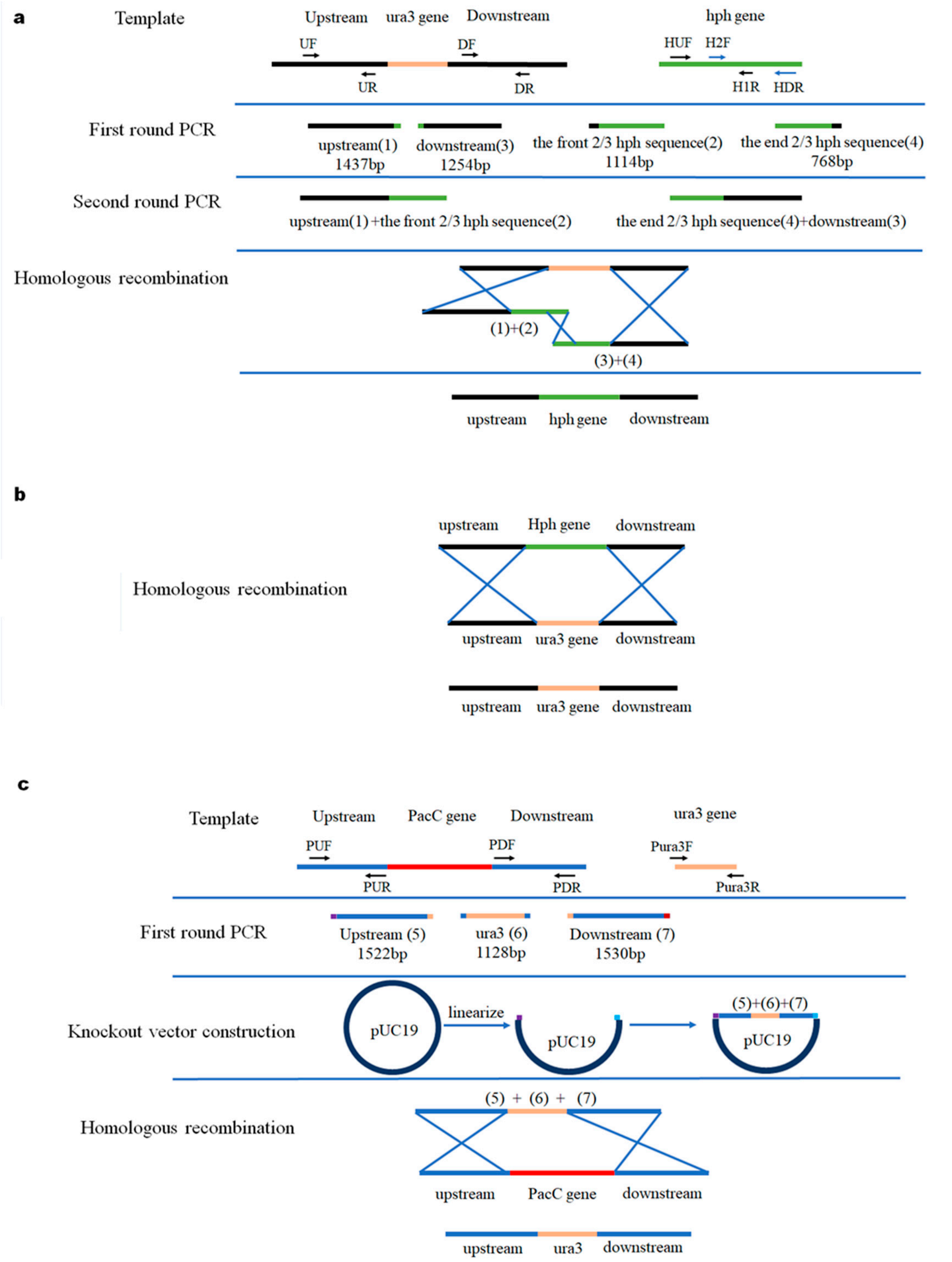

Suppl. Fig. S2. Flow chart of *Ijura3* and *IjpacC* gene knockout in *I. javanica*. a. *Ijura3* gene deletion. b. *Ijura3* complementation. c. *IjpacC* gene deletion.

Suppl. Fig. S3.

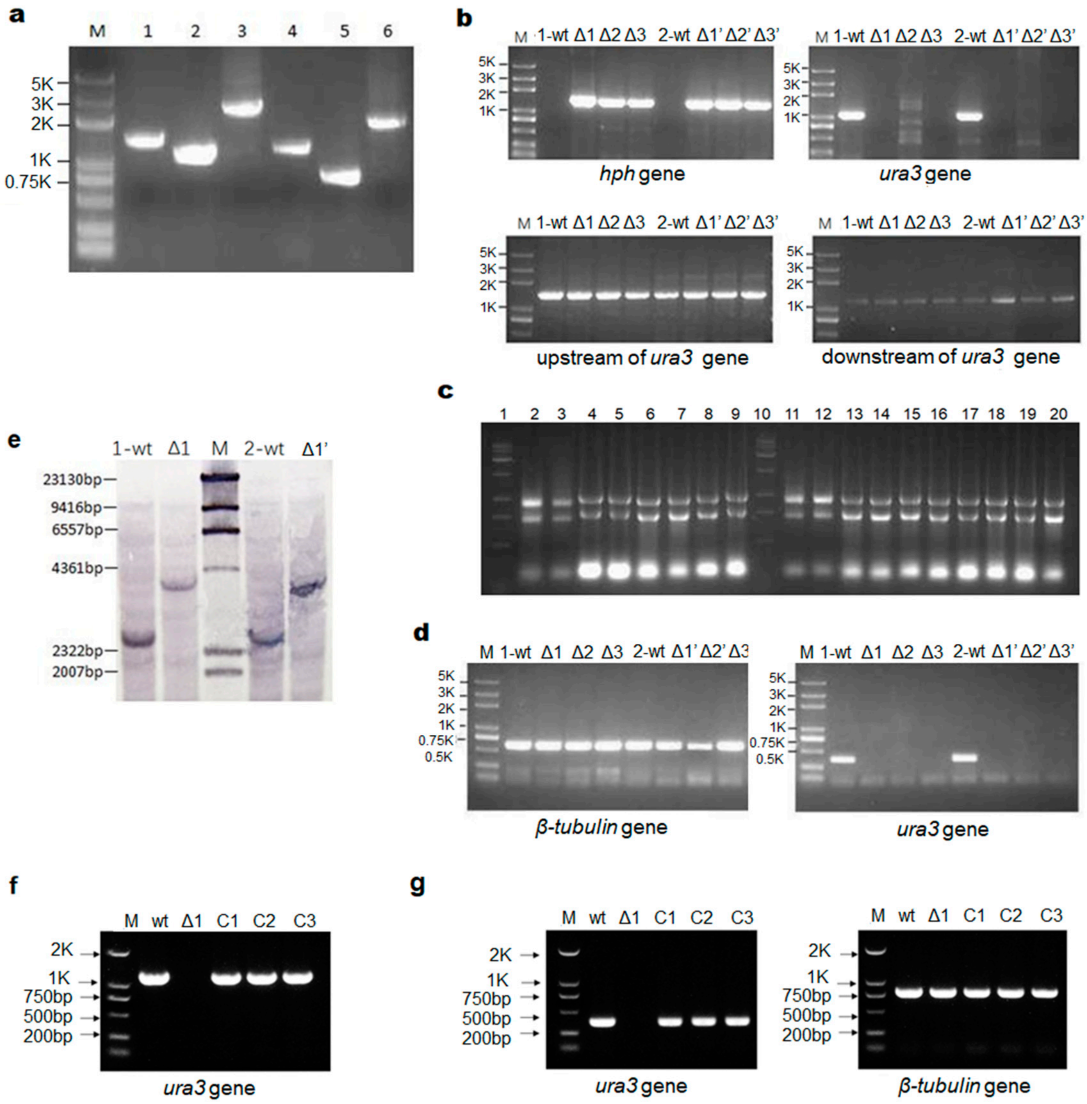

Suppl. Fig. S3. Confirmation of constructs and transformants of *ΔIjura3* mutants and complements (*ΔIjura3::ura3*). a. Electrophoresis detection of PCR products. M: marker; lane 1, upstream of *ura3* gene; lane 2, fore-2/3 fragment of hygromycin resistant gene; lane 3: fusion fragment of lane 1 & 2; lane 4, downstream of *ura3* gene; lane 5, post-2/3 fragment of hygromycin resistant gene; lane 6: fusion fragment of lane 4 & 5. b. PCR detection of positive transformants. M: marker; 1-wt and 2-wt are the wild type strains isolated from *Spodoptera litura* (Pj01) and from *Bemisia tabaci* (IJB01), respectively; *Δ*1, *Δ*2, *Δ*3 are three ura3 mutants from the strain Pj01, and *Δ*1', *Δ*2', *Δ*3' are three mutants from the strain IJB01. c. Electrophoresis detection of RNA integrity. Lane 1 & 10, marker; lane 2,3,11,12, the wild-type strain; lane 4-9, 13-20, transformants. d. RT-PCR detection of positive transformants. e. Southern blot analysis. A probe of 547-bp fragment (within the upstream flanking sequence of the ura3 gene) was hybridized with the genomic DNA digested with *Nae* I. A fragment

of ~4000 bp in size was detected in the *Ijura3* mutant strain, which including the upstream and downstream sequences of *ura3* gene, and the hygromycin resistant gene (*hph*). A fragment of ~2660 bp in size was detected in the wild-type strain, which including the ura3 gene and its upstream flanking sequence. M. λ-*Hind*III digestion marker. f. PCR detection of *ura3* complements. g. RT-PCR detection of *ura3* complements. C1, C2, C3 are three complements.
